# Noninvasive Screening for Elevated LVEDP and Health Status in Outpatients at Risk for Heart Failure

**DOI:** 10.1016/j.jacadv.2025.102002

**Published:** 2025-07-18

**Authors:** Omar Cantu-Martinez, Andrew A. Girard, Weiwei Jin, Derek Rinderknecht, Thomas Cheek, John A. Spertus

**Affiliations:** a University of Missouri-Kansas City's Healthcare Institute for Innovations in Quality, Kansas City, Missouri, USA; b Saint Luke's Mid America Heart Institute, Kansas City, Missouri, USA; c Ventric Health, Pasadena, California, USA; d King's College London, London, United Kingdom

**Keywords:** early detection, heart failure, health status, KCCQ-12, screening

## Abstract

**Background:**

Heart failure (HF) is frequently underrecognized in primary care due to nonspecific symptoms, resulting in many patients presenting with severely compromised health status (symptoms, function, and quality of life) at the time of diagnosis.

**Objectives:**

The purpose of this study was to evaluate the diagnostic yield of screening outpatients at risk for HF using a noninvasive assessment of left ventricular end-diastolic pressure (LVEDP) and describe the health status of patients identified with elevated LVEDP.

**Methods:**

A convenience sample of adults with diabetes mellitus, chronic kidney disease (CKD), or suspected HF were screened at 3 primary care clinics using the Vivio System to identify patients with LVEDP >18 mm Hg (positive screening). Among patients with a positive screening result, health status was assessed using the Kansas City Cardiomyopathy Questionnaire Overall Summary (KCCQ-OS) score.

**Results:**

Among 2,040 screened patients (mean age 74 ± 8 years; 49.8% women; 64.6% with diabetes mellitus; and 34.9% with CKD), 38.5% had an elevated LVEDP. Older patients, women, and those with CKD were more likely to have an elevated LVEDP (*P* < 0.01 for all). Of 653 KCCQ-OS scores collected (mean 85 ± 20), 31.4% had a KCCQ-OS of 100 (asymptomatic), and 26.5% had a KCCQ-OS <80, consistent with NYHA functional class II-IV.

**Conclusions:**

Nearly 40% of screened patients with HF risk factors had an elevated LVEDP, with over two-thirds reporting significant health status impairment. Combining the KCCQ with noninvasive LVEDP assessments can identify patients who may require further HF evaluation. Future studies should assess the impact of these strategies on patients' subsequent treatment, health status, and clinical events.

Multiple heart failure (HF) therapies have been shown to reduce clinical events and improve patients' health status (their symptoms, function, and quality of life).^1^ With the prevalence of HF continuing to increase, projected to affect 8.5 million Americans by 2030,^2^ there is a pressing need to improve its diagnosis and treatment. However, diagnosing HF in primary care can be challenging, particularly among elderly patients with multiple comorbidities.^3^, ^4^, ^5^ Factors contributing to delayed diagnosis include nonspecific presenting symptoms potentially attributed to age or comorbidities and the limited availability of effective HF screening modalities.^6^ Identifying HF earlier in the disease course could support initiating effective therapies to prevent HF progression, reduce mortality, and improve health status. This is particularly relevant for HF patients with preserved ejection fraction, given evolving evidence showing that sodium-glucose cotransporter-2 inhibitors, glucagon-like peptide 1 receptor agonists, and mineralocorticoid receptor antagonists prevent death and HF hospitalizations, while also improving patients' health status.^7^, ^8^, ^9^

In an attempt to improve the early recognition and treatment of HF, previous studies have evaluated the prevalence and clinical characteristics of undiagnosed HF in elderly outpatients using algorithms integrating clinical, laboratory, and echocardiographic data.^3^^,^^5^ However, many of these measures are not routinely available in primary care settings, limiting their usefulness. Recently, the Vivio System was cleared by the U.S. Food and Drug Administration (FDA) to noninvasively screen for a left ventricular end-diastolic pressure (LVEDP) >18 mm Hg to improve early detection of HF.^10^ This report describes the diagnostic yield of the Vivio System in 3 primary care outpatient clinics and, among those with an estimated elevated LVEDP, their HF-specific health status at the time of diagnosis. These real-world, descriptive results can provide the foundation for future studies testing a novel opportunity to recognize and treat the growing population of patients with HF.

## Methods

### Study population and design

The data in this cross-sectional report came from a convenience sample of the first 3 sites that implemented the Vivio System (Ventric Health) in routine clinical practice. Data were collected between August 13, 2024, and November 11, 2024. These sites were primary care practices that adopted a screening protocol using the Vivio System in adults at higher risk for HF due to a history of diabetes mellitus (DM); a history of chronic kidney disease (CKD) stage ≥3; or a physician's clinical suspicion for HF; the presumed rationale when screening was done in patients without either DM or CKD. Patients were excluded if they had a known diagnosis of HF or had contraindications to LVEDP assessment with the Vivio System (intravascular access or therapy, arteriovenous shunt/fistula on the affected upper extremity, mastectomy or lymph node dissection on the procedure's affected upper extremity, and any implantable electrical cardiac device, including permanent pacemakers). Patients who were screened with the Vivio System and found to have an estimated elevated LVEDP (positive screening) were offered, but not required to complete, the 12-item Kansas City Cardiomyopathy Questionnaire (KCCQ) to assess their health status.^11^ This study was reviewed and approved by the Saint Luke's Institutional Review Board (SLHS IRB Number: SLHS-25-008). The analysis was conducted using deidentified data obtained from routine clinical care, and patient-level consent was not required. The Vivio System results and KCCQ scores were stored in a database maintained by Ventric Health. All data were designed and analyzed in collaboration with Ventric Health, and interpretation was performed collaboratively by all coauthors.

### Vivio System

The Vivio System (Ventric Health) is a 510k FDA cleared device that can identify an elevated LVEDP noninvasively. The device incorporates 2 separate components, a modified pneumatic brachial blood pressure cuff and a synchronized single-lead electrocardiogram. Following inflation, the blood pressure cuff collects standard blood pressure measurements (systolic, diastolic, and mean arterial blood pressure) and then reinflates to a suprasystolic blood pressure (systolic blood pressure +35 mm Hg) to collect 40 seconds of brachial pulse waveform and electrocardiogram data. Estimated elevated LVEDP (>18 mm Hg) is then identified using a classification model with a reported sensitivity and specificity of 80% and 83%, respectively.^10^

### Health status assessments

The KCCQ is a patient-reported outcome measure quantifying the symptoms, physical and social functioning, and quality of life of patients with HF. While each domain is assessed independently, they can be combined into an Overall Summary (OS) score to quantify the full impact of HF on patients' health status. To increase its feasibility in routine clinical care, the original 23-item KCCQ was shortened to a 12-item questionnaire (KCCQ-12) that retains the psychometric properties of the original version.^11^ Scores range from 0 to 100 points, with lower scores representing worse health status and higher scores representing better health status.^12^ Cross-sectional scores are often described in 25-point ranges from very poor to poor (0-24), poor to fair (25-49), fair to good (50-74), and good to excellent (75-100). While it is difficult to map KCCQ-12 score to the NYHA functional classification due to interphysician variability in assigning the NYHA,^13^^,^^14^ KCCQ-OS scores of 0 to 44, 45 to 59, 60 to 79, and ≥80 roughly correspond to NYHA functional classes IV, III, II, and I, respectively. The KCCQ-12 score was included as a part of the Vivio System and was recommended for those with an estimated elevated LVEDP to further characterize their symptom severity.

### Statistical analysis

Demographic and clinical data were compared between patients with and without an estimated elevated LVEDP. Patients aged over 90 years were imputed as 90 to comply with the Health Insurance Portability and Accountability Act guidelines. Body mass index (BMI) was categorized as underweight (BMI <18.5), healthy weight (BMI 18.5 to <25), overweight (BMI 25 to <30), class 1 obesity (BMI 30 to <35), class 2 obesity (BMI 35 to <40), and class 3 obesity (BMI ≥40). Data are presented as mean ± SD for continuous variables and counts (percentages) for categorical data. The Shapiro-Wilk test was used to assess whether continuous variables were normally distributed. Comparisons between the 2 groups were assessed using *t*-tests for continuous variables and chi-squared tests for categorical variables.

The characteristics of patients with available KCCQ-12 scores were described with respect to their overall scores as well as 4 separate groups based on the range of their KCCQ-OS score: 100, 80 to 99, 60 to 79, and <60, roughly corresponding to asymptomatic, and NYHA functional classes I, II, and III/IV, respectively. All data were analyzed using Python 3.9.13 with pandas 1.4.3 and scipy 1.13.1.

## Results

### Population characteristics and diagnostic yield among screened patients

The overall study cohort included 2,040 screened patients. The mean age was 74 ± 8 years; 1,015 (49.8%) were women, 796 (39.0%) had class 1 obesity or higher, 1,318 (64.6%) with a history of DM, and 711 (34.9%) had CKD, of whom 263 (12.9%) had both DM and CKD, and 274 (13.4%) had neither DM nor CKD and were presumably tested for suspicion of HF. Among screened patients, 785 (38.5%) had an estimated elevated LVEDP. Table 1 compares the patient characteristics of those with and without an estimated elevated LVEDP. Patients with an estimated elevated LVEDP were older (75 ± 8 vs 74 ± 8, *P* = 0.003), more often women (62.2% vs 42.0%, *P* < 0.001), and more likely to have CKD (38.3% vs 32.7%, *P* = 0.010).

**Table 1:** Patient Characteristics Between Those With Estimated Elevated LVEDP and Normal LVEDP

	Overall (N = 2,040)	Elevated LVEDP (n = 785, 38.5%)	Normal LVEDP (n = 1,255, 61.5%)	*P* Value
Demographic and clinical characteristics				
Age, mean (SD)^a^	74 (8)	75 (8)	74 (8)	0.003
Women, n (%)	1,015 (49.8%)	488 (62.2%)	527 (42.0%)	<0.001
BMI, mean (SD)	29 (6)	29 (6)	29 (6)	0.769
BMI categories, n (%)				0.634
Underweight - <18.5	42 (2.1%)	12 (1.5%)	30 (2.4%)	
Healthy weight - 18.5 to <25	413 (20.2%)	165 (21.0%)	248 (19.8%)	
Overweight - 25 to <30	789 (38.7%)	300 (38.2%)	489 (39.0%)	
Class 1 obesity - 30 to <35	528 (25.9%)	206 (26.2%)	322 (25.7%)	
Class 2 obesity - 35 to <40	184 (9.0%)	74 (9.4%)	110 (8.8%)	
Class 3 obesity - 40 or greater	84 (4.1%)	28 (3.6%)	56 (4.5%)	
Blood pressure, mean (SD)^b^				
Systolic blood pressure	135 (18)	145 (17)	129 (16)	<0.001
Mean arterial blood pressure	96 (13)	103 (12)	91 (11)	<0.001
Diastolic blood pressure	78 (12)	84 (11)	74 (11)	<0.001
Number of comorbidities, mean (SD)	1.6 (0.7)	1.6 (0.7)	1.6 (0.7)	0.197
Comorbidities, n (%)^c^				0.582
1	1,006 (49.3%)	399 (50.8%)	607 (48.4%)	
2	797 (39.1%)	303 (38.6%)	494 (39.4%)	
3	216 (10.6%)	75 (9.6%)	141 (11.2%)	
4	21 (1.0%)	8 (1.0%)	13 (1.0%)	
5	0 (0.0%)	0 (0.0%)	0 (0.0%)	
DM	1,318 (64.6%)	449 (57.2%)	869 (69.2%)	<0.001
DM without CKD	1,055 (51.7%)	357 (45.5%)	698 (55.6%)	<0.001
CKD	711 (34.9%)	301 (38.3%)	410 (32.7%)	0.010
CKD without DM	448 (22.0%)	209 (26.6%)	239 (19.0%)	<0.001
DM and CKD	263 (12.9%)	92 (11.7%)	171 (13.6%)	0.237
No DM nor CKD	274 (13.4%)	127 (16.2%)	147 (11.7%)	0.005
COPD	181 (8.9%)	65 (8.3%)	116 (9.2%)	0.507
Hypertension	1,023 (50.1%)	402 (51.2%)	621 (49.5%)	0.475
Arrhythmia	27 (1.3%)	10 (1.3%)	17 (1.4%)	>0.999
None of the above	72 (3.5%)	35 (4.5%)	37 (2.9%)	0.094

BMI = body mass index; CKD = chronic kidney disease; COPD = chronic obstructive pulmonary disease; DM = diabetes mellitus; LVEDP = left ventricular end-diastolic pressure.

a Patients with age ≥90 years were imputed as 90 as the exact age was not available due to HIPAA privacy rules.

b Brachial blood pressure values taken with the Vivio System are not currently cleared by FDA.

c Only counting hypertension, DM, COPD, CKD, and arrhythmia.

### Health status of patients with elevated LVEDP at the time of diagnosis

Among the 785 patients with an estimated elevated LVEDP, 653 (83.2%) completed the KCCQ-12. Table 2 describes patients' characteristics overall and stratifies them by the ranges of impairment in their health status. Almost a third (n = 205, 31.4%) had KCCQ-OS scores of 100, suggesting that they were asymptomatic (American Heart Association/American College of Cardiology stage B, pre-HF). Of those with American Heart Association/American College of Cardiology Stage C HF, 275 (42.1%) had scores of 80 to 99 (consistent with NYHA functional class I), 94 (14.4%) had scores of 60 to 79 (consistent with NYHA functional class II), and 79 (12.1%) had scores <60 (consistent with NYHA functional class III/IV) (Central Illustration).

**Table 2:** Patient Characteristics With Estimated Elevated LVEDP Across KCCQ-OS Score Groups

	Overall (N = 653)	KCCQ-OS Score			
		100 (n = 205, 31.4%)	80-99 (n = 275, 42.1%)	60-79 (n = 94, 14.4%)	<60 (n = 79, 12.1%)
Demographic and clinical characteristics					
Age, mean (SD)^a^	75 (8)	74 (8)	75 (7)	76 (9)	74 (9)
Women, n (%)	400 (61.3%)	113 (55.1%)	172 (62.5%)	64 (68.1%)	51 (64.6%)
BMI, mean (SD)	29 (6)	28 (6)	29 (5)	29 (5)	32 (7)
BMI categories, n (%)					
Underweight - <18.5	10 (1.5%)	4 (2.0%)	3 (1.1%)	1 (1.1%)	2 (2.5%)
Healthy weight - 18.5 to <25	135 (20.7%)	47 (22.9%)	62 (22.5%)	17 (18.1%)	9 (11.4%)
Overweight - 25 to <30	252 (38.6%)	91 (44.4%)	99 (36.0%)	42 (44.7%)	20 (25.3%)
Class 1 obesity - 30 to <35	167 (25.6%)	39 (19.0%)	79 (28.7%)	26 (27.7%)	23 (29.1%)
Class 2 obesity - 35 to <40	66 (10.1%)	20 (9.8%)	24 (8.7%)	6 (6.4%)	16 (20.3%)
Class 3 obesity - 40 or greater	23 (3.5%)	4 (2.0%)	8 (2.9%)	2 (2.1%)	9 (11.4%)
Blood pressure, mean (SD)^b^					
Systolic blood pressure	144 (17)	144 (16)	144 (16)	146 (22)	143 (16)
Mean arterial blood pressure	103 (11)	103 (11)	102 (11)	103 (14)	102 (11)
Diastolic blood pressure	83 (11)	84 (11)	82 (10)	84 (12)	82 (10)
Number of comorbidities, mean (SD)	1.7 (0.7)	1.7 (0.7)	1.6 (0.7)	1.6 (0.7)	1.8 (0.8)
Comorbidities, n (%)^c^					
1	312 (47.8%)	98 (47.8%)	132 (48.0%)	50 (53.2.%)	32 (40.5%)
2	265 (40.6%)	83 (40.5%)	112 (40.7%)	35 (37.2%)	35 (44.3%)
3	68 (10.4%)	21 (10.2%)	28 (10.2%)	9 (9.6%)	10 (12.7%)
4	8 (1.2%)	3 (1.5%)	3 (1.1%)	0 (0%)	2 (2.5%)
5	0 (0%)	0 (0%)	0 (0%)	0 (0%)	0 (0%)
Hypertension	342 (52.4%)	111 (54.1%)	146 (53.1%)	43 (45.7%)	42 (53.2%)
Diabetes mellitus	387 (59.3%)	127 (62.0%)	152 (55.3%)	55 (58.5%)	53 (67.1%)
COPD	55 (8.4%)	13 (6.3%)	22 (8.0%)	6 (6.4%)	14 (17.7)
Chronic kidney disease	254 (38.9%)	70 (34.1%)	119 (43.3%)	39 (41.5%)	26 (32.9%)
Arrhythmia	9 (1.4%)	4 (2.0%)	3 (1.1%)	1 (1.1%)	1 (1.3%)
None of the above	31 (4.7%)	14 (6.8%)	10 (3.6%)	3 (3.2%)	4 (5.1%)
KCCQ scores, mean (SD)					
Overall summary score	85 (20)	100 (0)	91 (20)	71 (5)	41 (14)
Physical limitation	87 (29)	100 (0)	94 (20)	72 (32)	43 (28)
Symptom frequency	88 (19)	100 (0)	93 (9)	77 (14)	50 (22)
Quality of life	79 (27)	100 (0)	82 (19)	60 (18)	33 (23)
Social limitation	87 (38)	100 (0)	97 (30)	78 (35)	38 (27)

KCCQ-OS = Kansas City Cardiomyopathy Questionnaire Overall Summary; other abbreviations as in Table 1.

a Patients with age ≥90 years were imputed as 90 as the exact age was not available due to HIPAA privacy rules.

b Brachial blood pressure values taken with the Vivio System are not currently cleared by FDA.

c Only counting hypertension, DM, COPD, CKD, and arrhythmia.

**Central Illustration:**
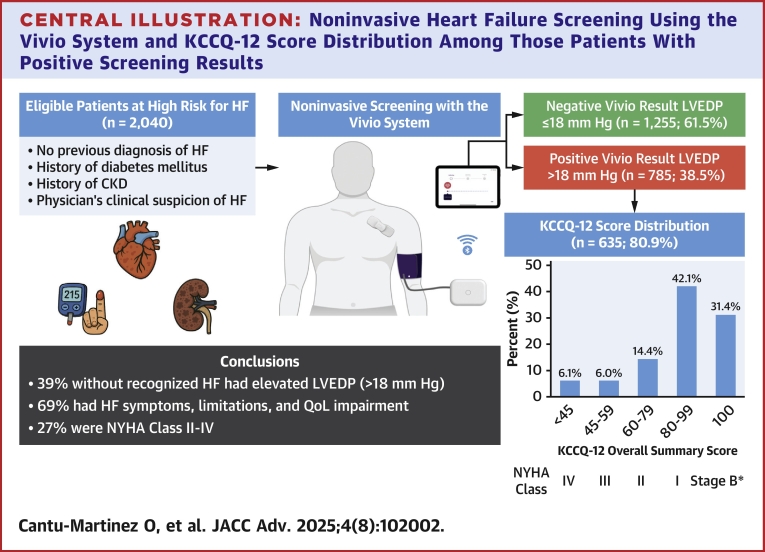
Noninvasive Heart Failure Screening Using the Vivio System and KCCQ-12 Score Distribution Among Those Patients With Positive Screening Results High-risk outpatients identified based on a history of diabetes mellitus, chronic kidney disease stage ≥3, or physician's clinical suspicion of heart failure (HF), underwent noninvasive screening using the Vivio System. Patients were stratified by estimated left ventricular end-diastolic pressure (LVEDP), with a threshold of >18 mm Hg indicating a positive result. Bar chart depicting the distribution of Kansas City Cardiomyopathy Questionnaire (KCCQ) scores, among patients with estimated elevated LVEDP >18 mm Hg. ∗Asymptomatic AHA/ACC Stage B. ACC = American College of Cardiology; AHA = American Heart Association; CKD = chronic kidney disease; HF = heart failure; KCCQ = Kansas City Cardiomyopathy Questionnaire; LVEDP = left ventricular end-diastolic pressure; QoL = quality of life.

## Discussion

Despite the development of numerous interventions to improve the outcomes of patients with HF, these therapies cannot be offered to those who are not diagnosed. Given that patients with HF are often elderly and have multiple comorbidities, it can be difficult to recognize HF in patients presenting to primary care. To help close this gap in care, the Vivio System was cleared by the FDA as a screening tool for HF to identify patients with estimated elevated LVEDP (>18 mm Hg). This initial report describes the diagnostic yield and health status burden of patients newly recognized with an estimated elevated LVEDP. More than one-third of the primary care patients screened had an estimated elevated LVEDP and, within this group, almost one-quarter reported substantial symptoms and impaired health status consistent with NYHA functional class II-IV and associated with higher risks of HF hospitalization and death.^15^

This study supports and extends the extant literature on screening high-risk patients for HF. The observed diagnostic yield in this study aligns well with prior efforts to screen elderly outpatients with comorbidities.^3^^,^^5^^,^^16^^,^^17^ One cross-sectional study aimed to assess the prevalence of unrecognized HF in patients over 65 years with dyspnea on exertion used echocardiography and biomarker measurements of N-terminal pro B-type natriuretic peptide and reported a prevalence of 15.7%.^3^ A separate study reported that about a quarter of residents in long-term care facilities had unrecognized HF.^5^ Another investigation among elderly patients with DM showed a 27.7% prevalence of previously undiagnosed HF.^16^ Furthermore, a multicenter prospective study found HF to be present in 39.2% of patients with DM.^17^ Collectively, these results are consistent with the present findings that screened higher-risk patients in primary care settings. Importantly, this study is the first to describe patients' health status at the time of potential HF recognition, showing that less than a third were asymptomatic and over a quarter had NYHA functional class II-IV symptoms.

DM and CKD are associated with a higher incidence of HF.^18^^,^^19^ Hence, incorporating these risk factors into a screening protocol likely increased the diagnostic yield of the Vivio System. These data suggest that a significant proportion of patients with cardio-renal and metabolic risk factors, but no formal HF diagnosis, have an elevated LVEDP. As such, additional diagnostic testing to characterize HF subtypes could be warranted to implement treatments that could improve their health status and prognosis.

It is notable that a higher proportion of screened women had elevated LVEDP, reinforcing previous research suggesting delayed HF diagnoses in women. Whether these delays help explain the lower use of medical therapy and poorer health status among women with known HF (compared with men) warrants further investigation.^20^, ^21^, ^22^ Should this hypothesis-generating concept prove true, it is possible that earlier and more systematic HF screening could reduce sex-based differences in care and outcomes.

Given the prevalence of elevated LVEDP in higher-risk patients, future studies to systematically assess the impact of routine HF screening on care and outcomes are needed. Researchers may consider hybrid implementation-effectiveness studies to understand how best to deploy such screening programs and their yield of confirmed HF diagnoses, use of guideline-recommended therapies, patients' health status outcomes, long-term clinical events, and the total cost of care. Moreover, assessing the impact of such programs on reducing sex-, race-, and socioeconomic-based disparities offer additional potential benefits of introducing novel screening strategies into routine care.

### Study limitations

The findings from this study should be interpreted in the context of several limitations. First, this study used a convenience sample from 3 primary care practices, which may limit generalizability to other populations. Specifically, the screening protocol focused on patients with an elevated baseline probability of HF (eg, a history of DM, CKD stage 3 or higher, or a clinical suspicion). Second, the completeness of screening among all eligible patients was not assessed, nor were the reasons for their primary care visits documented. Further investigation into these areas could inform future implementation strategies. Third, only medical conditions with a high prevalence in patients with HF (eg, hypertension, DM, and CKD) were recorded by the clinical staff, precluding the opportunity to refine potential screening criteria for LVEDP assessments. Fourth, this study was not designed to evaluate the operating characteristics of the Vivio System, such as intraindividual or interindividual variability, nor did it include comparisons with established diagnostic modalities such as natriuretic peptides or echocardiography. Additionally, HF was not independently confirmed among participants with estimated elevated LVEDP and/or HF symptoms. Future studies are needed describing the rate of agreement with other diagnostic testing, and changes in medications and outcomes resulting from earlier recognition. Such studies could further clarify the potential advantages of HF screening and its impact on subsequent care and outcomes.

## Conclusions

Considering the prevalence of HF, its evolving treatment options, and its impact on patients' lives, there is a clear need for novel strategies to improve its diagnosis in primary care. In this initial experience with the Vivio System, nearly 40% of patients meeting screening criteria had a positive result, with over two-thirds having significant health status impairment. Combining the KCCQ with noninvasive LVEDP assessment is a promising strategy to identify a significant number of patients who may benefit from further HF evaluation and treatment, potentially improving their health status and reducing clinical events.

Perspectives **COMPETENCY IN SYSTEMS-BASED PRACTICE:** The Vivio System, a novel HF screening tool, incorporates a patient-reported health status assessment to enable earlier detection and treatment of HF, with the potential to improve clinical outcomes.

**TRANSLATIONAL OUTLOOK:** The availability of novel, scalable tools like the Vivio System may facilitate broader implementation of systematic HF screening in clinical practice. Future studies should evaluate their impact on confirmed HF diagnosis rates, initiation of guideline-directed medical therapy, patient-reported health status, clinical outcomes, health care costs, and disparities across sex, race, and socioeconomic status.

## Funding support and author disclosures

Dr Cantu-Martinez has received funding from 10.13039/100000050NHLBI award number T32HL110837. Dr Girard has received funding from 10.13039/100000050NHLBI award number T32HL110837. Dr Jin discloses providing consultative services to and incentive compensation with Ventric Health. Dr Rinderknecht discloses providing consultative services to and incentive compensation with Ventric Health. Dr Cheek discloses providing consultative services to and incentive compensation with Ventric Health. Dr Spertus provides consultative services on patient-reported outcomes and evidence evaluation to Alnylam, Janssen, Bristol Myers Squibb, Terumo, Cytokinetics, BridgeBio, Ventric Health, and Imbria; holds research grants from the 10.13039/100000002National Institutes of Health, the Patient-Centered Outcomes Research Institute, the American College of Cardiology Foundation, Lexicon, Imbria, and Janssen; owns the copyright to the Seattle Angina Questionnaire, Kansas City Cardiomyopathy Questionnaire, and Peripheral Artery Questionnaire; and serves on the Board of Directors for Blue Cross Blue Shield of Kansas City.
